# Data supporting regulating temporospatial dynamics of morphogen for structure formation of the lacrimal gland by chitosan biomaterials

**DOI:** 10.1016/j.dib.2016.11.042

**Published:** 2016-11-19

**Authors:** Ya-Chuan Hsiao, Tsung-Lin Yang

**Affiliations:** a Department of Otolaryngology, National Taiwan University Hospital and College of Medicine, Taipei, Taiwan, ROC; b Department of Ophthalmology, Zhongxing Branch, Taipei City Hospital, Taipei, Taiwan, ROC; c Department of Ophthalmology, College of Medicine, National Yang-Ming University, Taipei, Taiwan, ROC; d Research Center for Developmental Biology and Regenerative Medicine, National Taiwan University, Taipei, Taiwan, ROC; e Graduate Institute of Clinical Medicine, College of Medicine, National Taiwan University, Taipei 10002, Taiwan, ROC

**Keywords:** Lacrimal gland, Chitosan, Branching morphogenesis, Hepatocyte growth factor

## Abstract

The lacrimal gland is responsible for tear synthesis and secretion, and is derived from the epithelia of ocular surface and generated by branching morphogenesis. The dataset presented in this article is to support the research results of the effect of chitosan biomaterials on facilitating the structure formation of the lacrimal gland by regulating temporospatial dynamics of morphogen. The embryonic lacrimal gland explants were used as the standard experimental model for investigating lacrimal gland branching morphogenesis. Chitosan biomaterials promoted lacrimal gland branching with a dose-dependent effect. It helped *in vivo* binding of hepatocyte growth factor (HGF) related molecules in the epithelial-mesenchymal boundary of emerging epithelial branches. When mitogen-activated protein kinase (MAPK) or protein kinase B (Akt/PKB) inhibitors applied, the chitosan effects reduced. Nonetheless, the ratios of MAPK and Akt/PKB phosphorylation were still greater in the chitosan group than the control. The data demonstrated here confirm the essential role of HGF-signaling in chitosan-promoted structure formation of the lacrimal gland.

**Specifications Table**

**Table:** 

Subject area	*Biology; Biomaterials*
More specific subject area	*The morphogenetic effect of chitosan biomaterials on the lacrimal gland structure formation*
Type of data	*Figures and Charts*
How data was acquired	*An ex vivo culture of the embryonic lacrimal gland explants was used.*
	*The ligands and receptors of HGF-related molecules were tested.*
Data format	*Raw and analyzed Data*
Experimental factors	*The lacrimal gland explants were cultured in a chitosan-containing system to induce branching morphogenesis. The effect of morphogen was tested.*
Experimental features	*The effect of chitosan on branching of the lacrimal gland explants was determined by imaging and quantitative analyses.*
Data source location	*The National Taiwan University, Taipei, Taiwan*
Data accessibility	*Data is available with this article*

**Value of the data**

• The data allow other researchers to investigate the effect of biomaterial using the explants of embryonic lacrimal glands as an experimental model.
• The data reveals the morphogenetic effects of chitosan in facilitating lacrimal gland structure formation.
• The chitosan-mediated morphogenetic effects on lacrimal gland explants originated from increasing expression and binding of hepatocyte growth factor (HGF) related molecules.

## 1 Experimental design, materials and methods

### 1.1 Preparation of the chitosan-containing system

To establish the chitosan-containing system for *ex vivo* culture of lacrimal gland explants, the water-soluble form of chitosan was firstly prepared. A 2 wt.% (w/v) chitosan solution was prepared by dissolving chitosan (Sigma–Aldrich Chemical Co. St. Louis, MO, USA) in 1 M acetic acid. The chitosan solution was then mixed with the medium used for lacrimal gland explant culture, neutralized with sodium hydroxide, added with additives, and prepared in the concentrations ranging from 0.1 to 0.4 mg/ml [1], [2]. For comparison, the mock was prepared similarly as that of the chitosan-containing medium, by adding the same amount of acetic acid and sodium hydroxide without chitosan. It had been confirmed that the mock and the control media had similar effects without significant differences in *ex vivo* explant morphogenesis [3]. It is therefore the control medium was used for comparison in all explant assays.

### 1.2 ex vivo explant culture of the lacrimal gland (lacrimal gland)

**Fig. 1:**
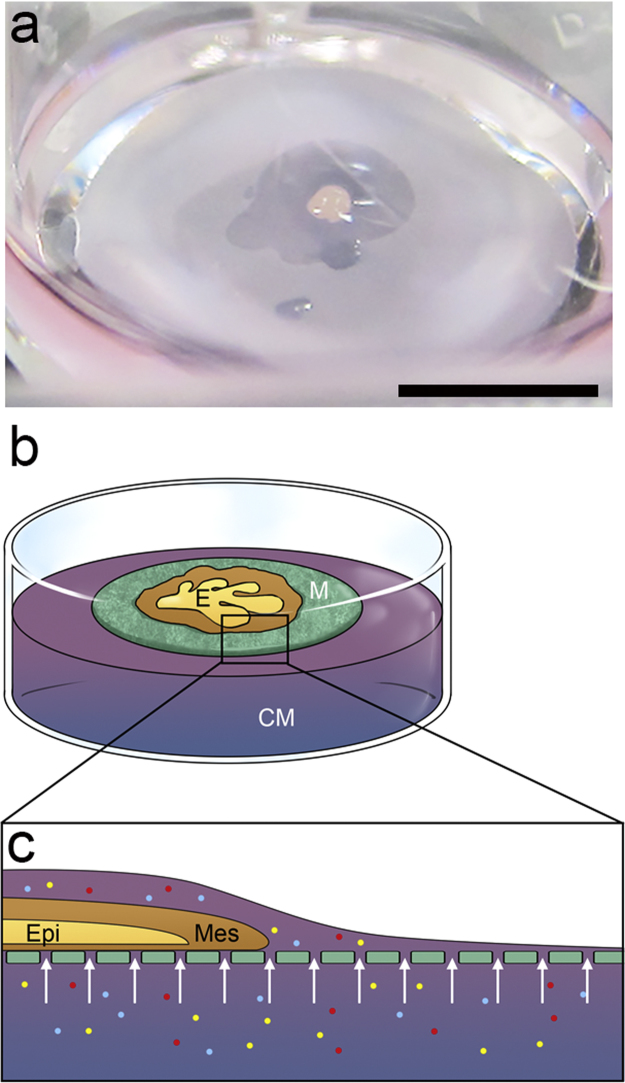
The *exvivo* chitosan-containing culture system for the structure formation of the lacrimal glands. (a) The chitosan system for the *ex vivo* culture experiments of the lacrimal gland explant. Scale bars: 5 mm. (b) A cartoon illustrates that the lacrimal gland explant is cultured on a membrane filter on the top of media. (E: explant; M: membrane; CM: culture media). (c) The lateral view of the cartoon demonstrates supply of culture medium components for explant culture and morphogenesis. (Epi: explant epithelia; Mes: explant mesenchyme; Small circles with colors represent distinct components of culture medium; Arrows: transportation of the components of culture medium).

In this study, the ICR mice strain was used. Animal protocols were approved by the Animal Care and Use Committee of the National Taiwan University and were in accordance with the guidelines. E16.5 lacrimal glands were used for explant culture, and the protocol followed the methods described previously [4]. The harvested lacrimal gland explants were placed on the 13 mm polycarbonate membrane filters (Nuclepore^®^, Whatman, Clifton, NJ, USA). The membrane had numerous tiny pores for transportation of the constituents of culture medium. The setting kept lacrimal gland explants cultured in a submerged fashion of culture medium with an air/medium interface **(**Fig. 1**).** The medium was composed of DMEM/F12 supplemented with 50 μg/ml ascorbic acid and 50 μg/ml transferrin. All explants were cultured at 37 °C in a humidified 5% CO_2_/95% air atmosphere. The cultured lacrimal gland explants were photographed and measured at the indicated time-points as experimental design. The lacrimal gland branching was quantified as the fold-change of branch numbers between the chitosan and control groups. At least three experimental repeats were averaged for comparison.

### 1.3 Modified ligand and carbohydrate engagement (LACE) assay

A modified ligand and carbohydrate engagement (LACE) assay was designed based on the methodology previously described [5]. The recombinant proteins of HGF and c-Met that were used as probes (R&D Systems, MN, USA). After culture in the chitosan and control groups, the lacrimal gland explants were harvested respectively from both groups, and then washed, permeabilized, and blocked with 10% BSA overnight at 4 °C. After the pretreatment, the lacrimal gland explants were incubated with indicated probes (50 nM) for three hours. In the preparation of HGF-c-Met probes, HGF was first incubated with c-Met for five minutes before use. The anti-human Fc antibody (R&D Systems, MN, USA) was used to detect the location of applied probes after fixation. Meanwhile, heparan sulfate proteoglycan (HSPG) was also stained in the lacrimal gland explants with anti-HSPG antibody (Chemicon, CA, USA), followed by the Cy dye-conjugated secondary antibody for visualization. The results were recorded by confocal microscopy (SP5; Leica) and analyzed [6].

### 1.4 Reverse transcription polymerase chain reaction (RT-PCR) and quantitative PCR

The lacrimal gland explants were harvested from each experimental condition. The RNA of lacrimal gland explants was extracted by RNeasy mini kit (Qiagen), and was prepared for synthesis of complementary DNA by ReverseAid kit (Thermo Fisher). The specific primers of HGF related genes used for RT-PCR and quantitative PCR were listed in Table 1. The experimental procedures of RT-PCR and quantitative PCR were performed as previously described [7].

### 1.5 Perturbation of HGF-related signaling pathways

Signaling perturbation of branching lacrimal gland explants was performed as previously described [8]. Protein inhibitors, including PD98059 (75 μM; Santa Cruz Biotechnology) and LY294002 (50 μM; Cayman Chemicals, Ann Arbor, MI) were used. The inhibitors were prepared with the concentrations suggested by manufacturers and published studies [9]. Meanwhile, the vehicles of protein inhibitors were applied with similar concentrations for comparison. The lacrimal gland explants were treated first by the inhibitors for 30 min, and then washed and replaced with fresh culture medium for subsequent culture. The suppressive effects of the signaling inhibitors were confirmed by Western blotting standardly as described [10].

## 2 Data

### 2.1 Data

**Fig. 2:**
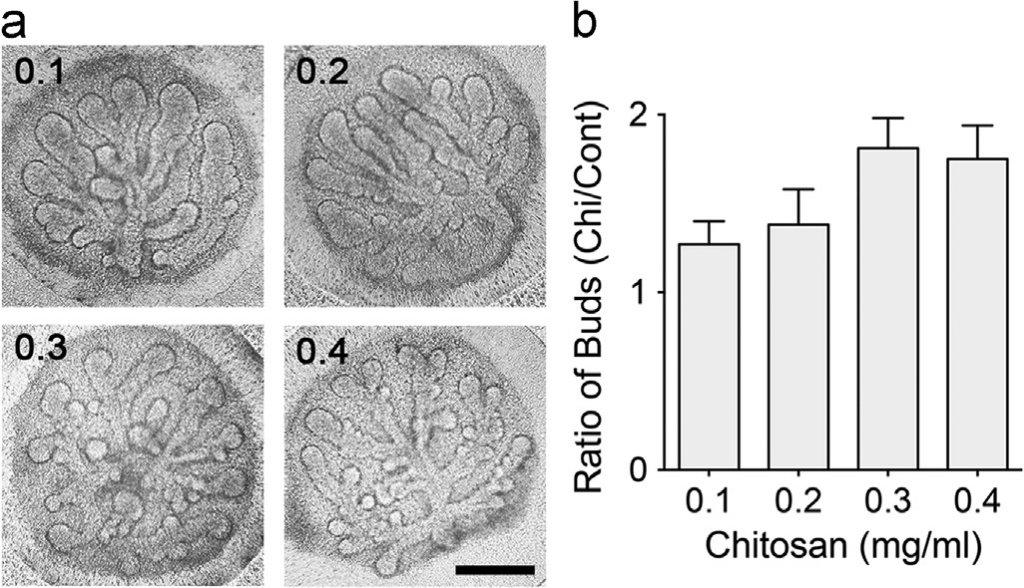
The dose-dependent effect of chitosan for the structure formation of the lacrimal glands. (a) The E16.5 lacrimal gland explants were cultured in the chitosan-containing system for 48 h with indicated concentrations (mg/ml). The scale bar=100 μm. (b) The quantitative analyses of branching number were presented as the ratio change of buds.

**Fig. 3:**
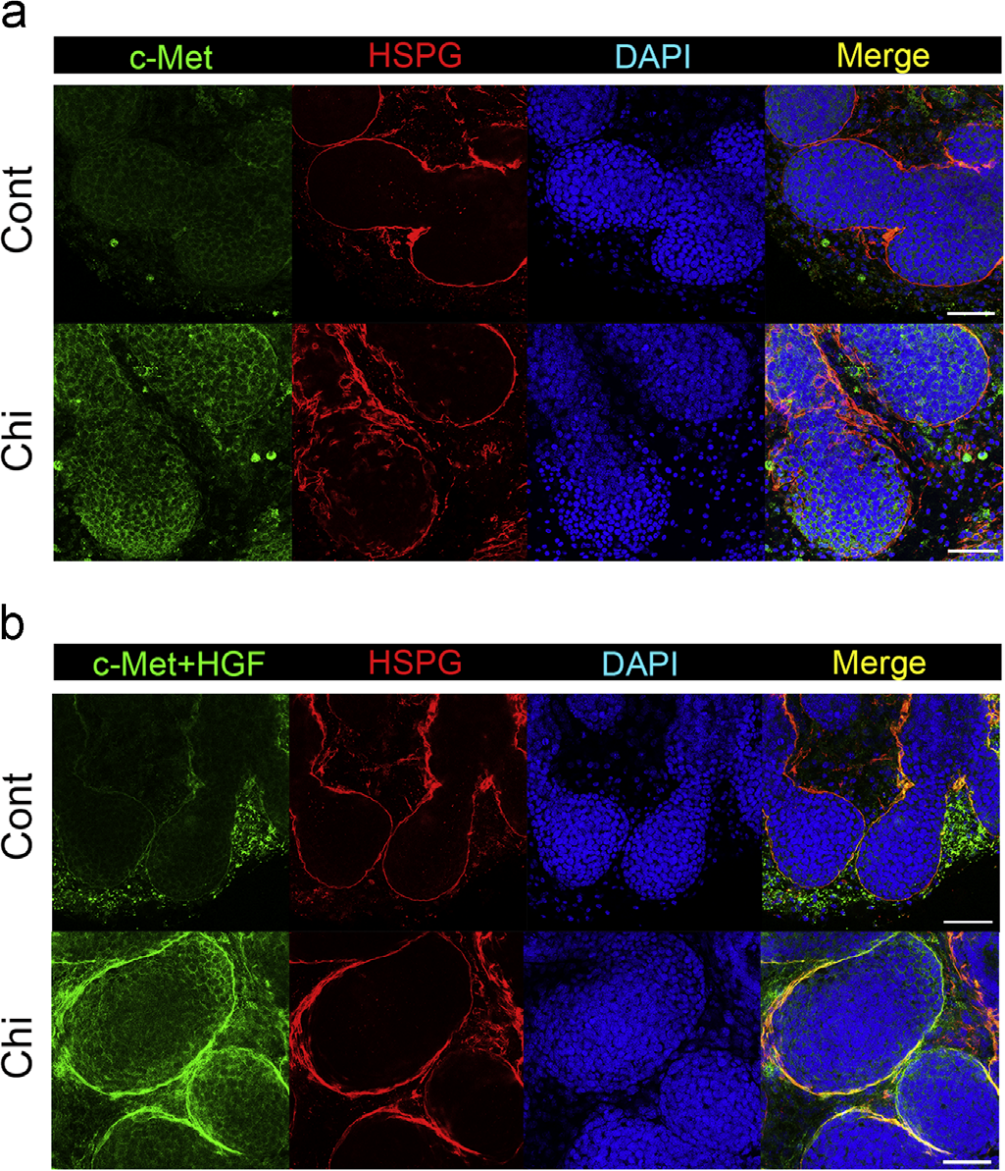
Alteration of *invivo* binding affinity of HGF-realted molecules by chitosan. The results of LACE assay with (a) c-Met, and (b) HGF-c-Met complex were demonstrated in the chitosan and control groups. HSPG delineated the basement membrane to show expression colocalization. (Cont: control group; Chi:chitosan groups; HSPG: heparan sulfate proteoglycan; DAPI: nucleus; Scale bar=50 μm).

**Fig. 4:**
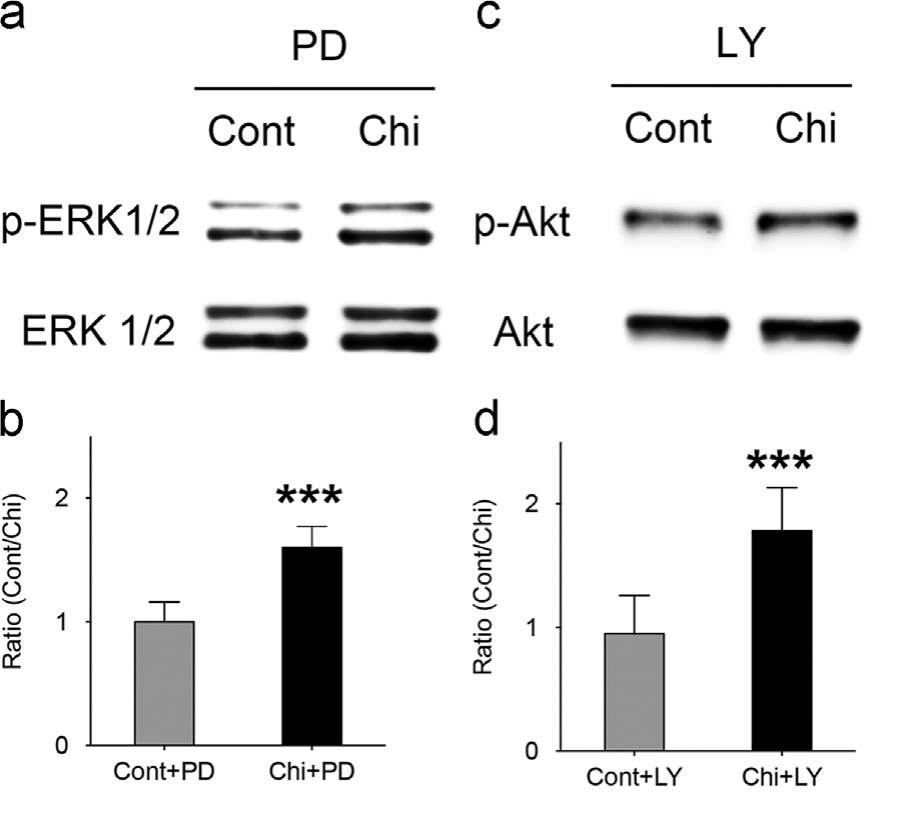
The effect of signaing transduction inhibitors was reduced in lacrimal gland explants cultured with chitosan. (a) Western blot analyses showed suppressed phosphorylation of MAPK pathway by PD98059 in both groups. (b) Quantitative analyses demonstrated the suppressive effect of PD98059 was greater in the control than in the chitosan groups. (c) Western blot analyses showed suppressive phosphorylation of the Akt/PKB pathway by LY294002 in both groups. (d) Quantitative analyses demonstrated the effect of LY294002 in the chitosan group reduced. (Cont: control group; Chi: chitosan group; Student׳s test, ***p<0.0001.).

The dataset of this article provides information to support the results in the article “regulating temporospatial dynamics of morphogen for structure formation of the lacrimal gland by chitosan biomaterials” [11]. Fig. 1, Fig. 2, Fig. 3, Fig. 4 show the experimental setting and results to confirm the regulation of temporospatial dynamics of morphogen in lacrimal gland explant system. Table 1 shows the primer sets used for molecular profiling.

### 2.2 Chitosan facilitates lacrimal gland branching morphogenesis in a dose-dependent manner

The morphogenetic effect of chitosan was evaluated in the e*x vivo* culture system of embryonic lacrimal gland explants. It is a standard model for studying branching morphogenesis of many organs [12], and has also been used to investigate the morphogenetic effect of chitosan [13], [14], [15]. The cultured lacrimal gland explants initiated branching morphogenesis in culture and continued through the whole culture period. Branching was promoted when chitosan was added in the culture system **(**Fig. 2**a).** When the concentration of chitosan increased from 0.1 to 0.3 mg/ml, it was found that branching ratio increased correspondingly. The tendency of increased branching number correlated with the concentration of chitosan, indicating that lacrimal gland branching morphogenesis was promoted by chitosan in a dose-dependent manner. Between the concentration 0.3 and 0.4 mg/ml of chitosan, no further increase of branching number was noted in the lacrimal gland explants. Accordingly, chitosan effect was most significant at a concentration of 0.3 mg/ml, which was then used for the following investigation of chitosan effects **(**Fig. 2**b)**[16], [17].

### 2.3 Chitosan enhances in vivo binding affinity of HGF-related molecules in cultured lacrimal gland explants

To confirm the effects of chitosan in facilitating *in vivo* binding of HGF-related molecules within the cultured lacrimal gland explants, the modified LACE assay for whole-mount experiments was performed on the cultured lacrimal gland explants in both control and chitosan groups. The ligand and receptors of HGF-related signaling were tested for endogenous binding affinity in lacrimal gland explants. The cultured lacrimal gland explants were first probed with c-Met in both groups **(**Fig. 3**a).** Since the ligand-receptor binding occurred along the emerging tips of epithelia, HSPG was co-stained to delineate the epithelial-mesenchymal boundary of cultured lacrimal gland explants. In addition, because the interaction between c-Met and HSPG was essential to conduct HGF signaling [18], the correlation between the localization of c-Met and HSPG provided spatial evidence of effective *in vivo* binding. Analyzing the confocal imaging in serial sections, it was found that expression of c-Met staining colocalized with that of HSPG **(**Fig. 3**a).** When both chitosan and control groups were compared, the intensity of c-Met was greater in the chitosan group than the control, and colocalized with HSPG. The phenomenon was obvious in the emerging tip of epithelia, where the branching was active. Similarly, when HGF-c-Met complexes were used as the probes, staining intensity in the chitosan group was greater than that of the control, including both probes. The staining appeared along the epithelial-mesenchymal boundary, matching the expression pattern of HSPG. The data showed that the *in vivo* affinity of HGF-related molecules was promoted by chitosan at the epithelial tip to promote branching structure of lacrimal gland explants **(**Fig. 3**b).**

### 2.4 Chitosan regulates downstream signaling of HGF to facilitate lacrimal gland branching morphogenesis

The specific inhibitors of the downstream pathways were applied to verify the blocking effects on lacrimal gland structural formation. PD98059 was widely used as an inhibitors of MAPK pathway [8]. When sub-lethal doses of PD were applied, no branching promoting effects were found. Similar phenomenon was observed when the lacrimal gland explants were treated with LY294002, the Akt/PKB inhibitor. The results confirmed the essential roles of HGF downstream signaling in chitosan morphogenetic effects. Nonetheless, comparing the results of both groups treated by the inhibitors in the Western blotting, the phosphorylation ratios of MAPK and Akt/PKB pathways were greater in the chitosan than the control groups **(**Fig. 4**).** Accordingly, HGF was not the only pathway to activate the downstream signaling of MAPK and Akt/PKB when chitosan was present.

## Figures and Tables

**Table 1:** Primers for RT-PCR and qPCR analyses.

Gene symbol	Primer sequences	Base pairs	Sequence accession number
GAPDH	F: TGGCATTGCTCTCAATGAC	122	NM_008084.2
	R: AGGGTTTCTTACTCCTTGG		
HGF	F: CTGAGGAATGTCACAGACTTC	276	NM_010427.5
	R: CCATGAATTTGACCTCTATGA		
c-Met	F: GCTACCAGTAAAGTGGATGG	377	NM_008591.2
	R: GGCAACAGAGAAGGATATGG		
HAI-1	F: GGGCAACAAGAACAACTACC	274	NM_016907.3
	R: CACAGTACCCTTTGTCACTG		
HAI-2	F: CTCTTCTGTCCTGAGTGTTC	544	NM_011464.2
	R: CAGCACTGGGAAACAAAGAC		
HGFa	F: CATGACCTTGTCTTGATCCG	242	NM_019447.2
	R: GTCAGCACCATATACCTCTG		
